# Identifying the differentially expressed peripheral blood microRNAs in psychiatric disorders: a systematic review and meta-analysis

**DOI:** 10.3389/fpsyt.2024.1390366

**Published:** 2024-05-17

**Authors:** Xiaoyan Liu, Liying Dong, Zhaowei Jiang, Mingfen Song, Pan Yan

**Affiliations:** ^1^ Department of Psychiatry, Affiliated Mental Health Center & Hangzhou Seventh People’s Hospital, Zhejiang University School of Medicine, Hangzhou, China; ^2^ Internal Medicine of Traditional Chinese Medicine, The 4th Clinical Medical College, Zhejiang Chinese Medical University, Hangzhou, China; ^3^ Molecular Biology Laboratory, Affiliated Mental Health Center & Hangzhou Seventh People’s Hospital, Zhejiang University School of Medicine, Hangzhou, China

**Keywords:** psychiatric disorders, microRNAs, biomarkers, systematic review, meta-analysis

## Abstract

**Background:**

Evidence has suggested that microRNAs (miRNAs) may play an important role in the pathogenesis of psychiatric disorders (PDs), but the results remain inconclusive. We aimed to identify specific differentially expressed miRNAs and their overlapping miRNA expression profiles in schizophrenia (SZ), major depression disorder (MDD), and bipolar disorder (BD), the three major PDs.

**Methods:**

The literatures up to September 30, 2023 related to peripheral blood miRNAs and PDs were searched and screened from multiple databases. The differences in miRNA levels between groups were illustrated by the standardized mean difference (SMD) and 95% confidence interval (95% CI).

**Results:**

In total, 30 peripheral blood miRNAs were included in the meta-analysis, including 16 for SZ, 12 for MDD, and 2 for BD, each was reported in more than 3 independent studies. Compared with the control group, miR-181b-5p, miR-34a-5p, miR-195-5p, miR-30e-5p, miR-7-5p, miR-132-3p, miR-212-3p, miR-206, miR-92a-3p and miR-137-3p were upregulated in SZ, while miR-134-5p, miR-107 and miR-99b-5p were downregulated. In MDD, miR-124-3p, miR-132-3p, miR-139-5p, miR-182-5p, miR-221-3p, miR-34a-5p and miR-93-5p were upregulated, while miR-144-5p and miR-135a-5p were downregulated. However, we failed to identify statistically differentially expressed miRNAs in BD. Interestingly, miR-132-3p and miR-34a-5p were upregulated in both SZ and MDD.

**Conclusions:**

Our study identified 13 differentially expressed miRNAs in SZ and 9 in MDD, among which miR-132-3p and miR-34a-5p were upregulated in both SZ and MDD by systematically analyzing qualified studies. These miRNAs may be used as potential biomarkers for the diagnosis of SZ and MDD in the future.

**Systematic Review Registration:**

http://www.crd.york.ac.uk/PROSPERO, identifier CRD42023486982.

## 1 Introduction

Psychiatric disorders (PDs) are debilitating disease with unknown etiology and pathogenesis, characterized by the dysfunction of complex emotional and cognitive processes (1). Many patients with PDs require long-term treatment to maintain social function and prevent symptom relapse, causing heavy public health and economic burden (2). Schizophrenia (SZ), major depression disorder (MDD) and bipolar disorder (BD) are the three major PDs with high disability and lethality (3). SZ is the most severe PDs characterized by hallucinations, delusions, disturbed emotions, and social withdrawal, with a lifetime prevalence of approximately 1% worldwide (4). MDD is characterized by depressed mood and anhedonia, with a lifetime prevalence of 2-21% worldwide (5). BD is characterized by recurrent episodes of mania and depression, as well as impairments in cognitive performance, which occurs with a lifetime prevalence of 1-2% (6). Currently, the diagnosis of PDs mainly relies on patient’s statements and doctor’s subjective judgment of clinical symptoms, rather than on pathological and physiological indicators, and many PDs have overlapping symptoms, resulting in high rates of misdiagnosis and missed diagnosis. Thus, there is an urgent need to seek objective, effective, convenient and feasible early molecular diagnostic biomarkers for PDs.

Both genetic and environmental factors are thought to contribute to PDs (7). Epigenetic mechanisms, which combine genetic and environmental factors by translating the environmental information into a genetic code, have been reported to regulate pathways affecting PDs (8). Epigenetic mechanisms, which include DNA methylation, histone modification, and noncoding RNA (ncRNA), can regulate the gene expression without perturbation of DNA sequences (9). Among them, microRNAs (miRNAs), as a class of small ncRNA molecules, have been given great attention for their potential role in the etiology and pathophysiology of many diseases (10, 11). miRNAs negatively regulate gene expression at the post-transcriptional level by inhibiting translation and/or activating messenger RNAs (mRNAs) degradation through binding to the 3’-untranslated region (3’-UTR) of target mRNAs (12). miRNAs have strong cell and tissue specificity, and these specific expressions are not only the basis for its functional study, but also good disease markers. Evidence indicates that miRNAs regulate several aspects of neurodevelopment, including neurogenesis, neuronal differentiation, and synaptic plasticity through complex genetic networks (13).

Recent studies have revealed that disturbances in miRNAs may contribute to the etiology of SZ, MDD and BD, but there were conflicting results between these studies (10, 14), which may be due to differences in study design, small sample size, different specimen types. Therefore, the purpose of the present study was to comprehensively analyze the expression profiles of peripheral blood miRNAs associated with the pathogenesis or development of SZ, MDD and BD, and identify their specific differentially expressed miRNAs and their overlapping miRNAs expression profiles, so as to explore whether one or more miRNAs are promising biomarkers for their early diagnosis.

## 2 Materials and methods

The study protocol and registration information are available at http://www.crd.york.ac.uk/PROSPERO/ (registration number: CRD42023486982).

### 2.1 Search strategy

This study was followed by recommendations from the Preferred Reporting Items for Systematic Reviews and Meta-Analysis (PRISMA) guideline. Literature search was conducted using Cochrane Library, PubMed, Embase, Medline, Wanfang, CNKI, and Weipu for studies published from February 2007 to September 2023, investigating differentially expressed miRNAs in SZ, MDD, or BD patients versus controls. The Search was performed using the following key terms: (“microRNA” OR “miRNA” OR “miR”) AND (“psychiatric disorders” and “schizophrenia” OR “SZ” OR “major depressive disorder” OR MDD OR “bipolar disorder” OR BD). A manual search of reference lists from relevant articles was conducted to uncover more potential studies.

### 2.2 Eligibility criteria

Studies were included if they met the criteria below: 1) case-control studies; 2) studies on differential expression of peripheral blood miRNAs in SZ, MDD or BD; 3) the relative miRNA expression was detected by real-time quantitative polymerase chain reaction (RT-qPCR) or miRNA PCR panel or microarray or sequencing; 4) the mean and standard deviation (SD) of miRNA expression in the case group and control group could be obtained, or the relevant data could be used to calculate the above indicators. The exclusion criteria were as follows: 1) studies were not conducted in human subjects; 2) incomplete data; 3) duplicate data; 4) reviews, meta-analyses, letters or conference.

### 2.3 Data extraction

Two authors independently manually screened and extracted the data from included studies. Any inconsistencies were discussed with a third author until consensus was reached. The following items for each included study were extracted: 1) first author; 2) year of publication; 3) country; 4) specimen type; 5) sample size; 6) age; 7) miRNA detection methods; 8) mean and SD of the identified miRNAs in each group. If the mean and SD couldn’t be extracted from studies, we tried to contact their authors. The studies we didn’t receive a response were listed in **Supplementary Table 1**. If different specimen types were involved in the same study, data extraction and corresponding analysis were performed separately.

### 2.4 Quality assessment

The quality of included studies was assessed by using the Newcastle–Ottawa Scale (NOS) (15), which consists of three dimensions: selection, comparability and exposure. The studies with a score ≥ 5 are regarded as high quality.

### 2.5 Target gene prediction and functional enrichment analysis

TargetScan and miRanda were used to predict the target genes for common differentially expressed miRNAs in SZ, MDD or BD. TargetScan algorithms eliminated genes with context scores < 50%. miRanda algorithms eliminated genes with maximum energy > -10. Genes co-identified by both databases were potential target genes for a given miRNA. Functional enrichment analysis of the predicted target genes was implemented with kyoto encyclopedia of genes and genomes (KEGG). We performed enrichment analysis with the cut-off criterion of *P* < 0.05.

### 2.6 Statistical analysis

All analyses were conducted by Stata 12.0. The standard mean difference (SMD) and its 95% confidence interval (CI) were used to combine the miRNA expression results. The between-study heterogeneity was evaluated by a Cochran’s Q-statistic and quantified by I^2^ metric value. If I^2^<50% and *P*>0.10, the fixed-effects model was conducted, otherwise, the random-effects model was applied. Subgroup analyses were performed based on specimen types. The potential for publication bias was examined by Begg’s test and Egger’s test. Leave-one-out sensitivity analysis was performed to detect the stability of the results. *P*<0.05 was considered statistically significant.

## 3 Results

### 3.1 Characteristics of eligible studies

**Figure 1:**
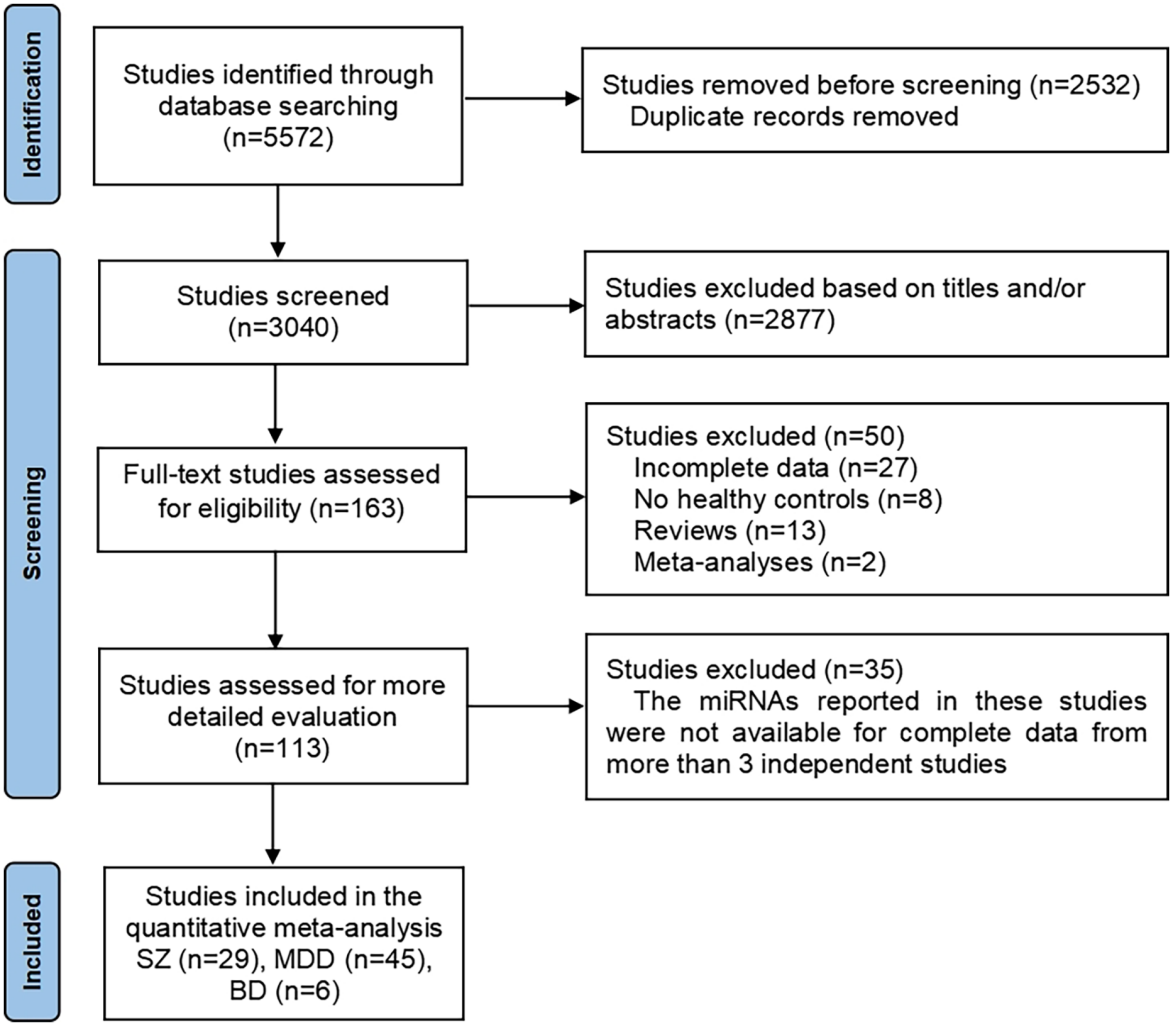
Flowchart of study selection process in this meta-analysis.

According to the search strategy, 5572 studies were identified in the database. After an initial screen, 2532 duplicate studies were removed. Next, 2877 studies were excluded based on titles and/or abstracts. The remaining 163 studies were evaluated in detail of which 50 studies were excluded due to incomplete data (n=27), no healthy controls (n=8), reviews (n=13) and meta-analysis (n=2). Of the remaining 113 studies, 35 were not included in the meta-analysis after data extraction because the mean and SD of the miRNAs reported in these studies could not be extracted from more than 3 independent studies (**Supplementary Table 2**). Finally, 78 studies were suitable for quantitative meta-analysis, of which 29 were identified for SZ (16–44), 45 for MDD (45–89), and 6 for BD (21, 24, 90–93). 2 studies (21, 24) were for both SZ and BD. The flowchart of the study screening process was shown in **Figure 1**. The NOS results showed that all the included studies were of high quality, with scores ranging from 5 to 9. The characteristics of the eligible studies were summarized in **Table 1**.

**Table 1:** Characteristics of eligible studies included in the meta-analysis.

Disease	Study	Country	Sample size		Specimen type	Detection method	NOS
			Case	Control			
SZ	Gardiner 2012 (16)	Australian	57	34	PBMC	qPCR	5
	Wang 2012 (17)	China	40	40	Plasma	qPCR	8
	Zhang 2014 (18)	China	60	72	Plasma/PBMC	qPCR	9
	Song 2014 (19)	China	20	20	Plasma	qPCR	9
	Sun 2015 (20)	China	25	13	Plasma/PBMC	qPCR	9
	Feng 2016 (21)	China	90	90	PBMC	qPCR	7
	Su 2017 (22)	China	174	80	Plasma/PBMC	qPCR	7
	Liu 2017 (23)	China	39	50	PBMC	qPCR	9
	Peng 2017 (24)	China	90	90	Plasma	qPCR	7
	Qu 2017 (25)	China	40	40	PBMC	qPCR	8
	Ma 2018 (26)	China	44	44	Whole blood	qPCR	6
	Bao 2018 (27)	China	46	49	PBMC	qPCR	9
	Feng 2018 (28)	China	90	90	Plasma	qPCR	7
	Fu 2018 (29)	China	17	16	Plasma	qPCR	9
	Wang 2019 (30)	China	35	15	Plasma/PBMC	qPCR	9
	Shi 2019 (31)	China	75	70	Plasma	qPCR	9
	Du 2019 (32)	China	49/100	46/100	Blood exosome	Sequencing/qPCR	8
	Guan 2021 (33)	China	40	40	Plasma	qPCR	9
	Lu 2021 (34)	China	26	48	PBMC	qPCR	9
	Jiang 2021 (35)	China	50	30	PBMC	qPCR	9
	Zhang 2021 (36)	China	150	150	Serum	qPCR	8
	Chen 2021 (37)	China	104	100	Plasma	qPCR	7
	Gou 2021 (38)	China	123	50	Whole blood	qPCR	8
	Pan 2021 (39)	China	118	47	Whole blood	qPCR	8
	Fu 2022 (40)	China	32	48	PBMC	qPCR	6
	Lu 2022 (41)	China	51	51	Serum	qPCR	9
	Huang 2022 (42)	China	92	89	Serum	qPCR	7
	Wang 2023 (43)	China	100	30	Serum	qPCR	9
	Jin 2023 (44)	China	51	51	Whole blood	qPCR	8
MDD	Rong 2012 (45)	China	42	40	Plasma	qPCR	9
	Belzeaux 2012 (46)	France	16	13	PBMC	qPCR	8
	Li 2013 (47)	China	40	40	Serum	qPCR	6
	Liu 2014 (48)	China	32	28	Plasma	qPCR	9
	Wan 2015 (49)	China	6	6	Serum	PCR Panel	8
	Camkurt 2015 (50)	Turkey	50	41	Plasma	qPCR	8
	Li 2015 (51)	China	18	18	Whole blood	qPCR	8
	Song 2015 (52)	China	36	30	Whole blood	qPCR	9
	Wang 2015 (53)	Sweden	169	52	Plasma	qPCR	7
	Gururajan 2016 (54)	Ireland	40	20	Whole blood	qPCR	6
	He 2016 (55)	China	32	30	PBMC	qPCR	8
	Liu 2016 (56)	China	62	73	Whole blood	qPCR	8
	Feng 2016 (57)	China	60	30	Plasma	qPCR	9
	Roy 2017 (58)	USA	18	17	Serum	qPCR	6
	Kolshus 2017 (59)	Ireland	7	21	Whole blood	qPCR	8
	Fang 2018 (60)	China	45	32	Plasma	qPCR	9
	Kuang 2018 (61)	China	84	78	Serum	qPCR	9
	Wang 2018 (62)	China	20	20	Whole blood	qPCR	7
	Gheysarzadeh 2018 (63)	Iran	39	36	Serum	qPCR	8
	Yuan 2018 (64)	China	100	120	Serum	qPCR	8
	Hung 2019 (65)	China	84	43	PBMC	qPCR	9
	Zhu 2019 (66)	China	90	60	Serum	qPCR	5
	Lv 2019 (67)	China	59	59	Serum	qPCR	9
	Tian 2019 (68)	China	104	52	Serum	qPCR	9
	Zhao 2019 (69)	China	97	63	Plasma	qPCR	9
	Kong 2019 (70)	China	27	46	Whole blood	qPCR	9
	Meng 2020 (71)	China	50	50	Serum	qPCR	6
	Fu 2020 (72)	China	59	59	Serum	qPCR	9
	Cao 2020 (73)	China	63	63	Serum	qPCR	9
	Liang 2020 (74)	China	30	30	Serum exosome	qPCR	7
	Xu 2020 (75)	China	41	31	PBMC	qPCR	7
	Qian 2020 (76)	China	45	32	Plasma	qPCR	7
	Wei 2020 (77)	China	33	46	Blood exosome	qPCR	6
	Ding 2021 (78)	China	50	50	Whole blood	qPCR	6
	Al-Rawaf 2021 (79)	Saudi Arabia	40	30	Serum	qPCR	8
	Hung 2021 (80)	China	52	31	Serum exosome	qPCR	9
	Liu 2021 (81)	China	20	20	Serum	qPCR	6
	He 2021 (82)	China	40	34	Plasma	qPCR	8
	Roumans 2021 (83)	Sweden	50	49	Plasma	qPCR	7
	Zhao 2021 (84)	China	77	80	Serum	qPCR	8
	Xian 2022 (85)	China	6	3	Serum exosome	qPCR	7
	Lin 2022 (86)	China	216	200	Serum	qPCR	9
	Brás 2023 (87)	Portugal	32	40	PBMC	qPCR	7
	Deng 2023 (88)	China	113	107	Serum exosome	qPCR	8
	Wu 2023 (89)	China	24	24	Serum	qPCR	6
BD	Rong 2011 (90)	China	21	21	Plasma	qPCR	8
	Feng 2016 (21)	China	90	90	PBMC	qPCR	7
	Peng 2017 (24)	China	90	90	Plasma	qPCR	7
	Xu 2018 (91)	China	105	100	Plasma	qPCR	9
	Camkurt 2020 (92)	Turkey	58	51	Whole blood	qPCR	8
	Tekdemir 2022 (93)	Turkey	66	66	Whole blood	qPCR	8

SZ, schizophrenia; MDD, major depression disorder; BD, bipolar disorder; PBMC, peripheral blood mononuclear cell.

### 3.2 Main results and sub-group analysis

**Figure 2:**
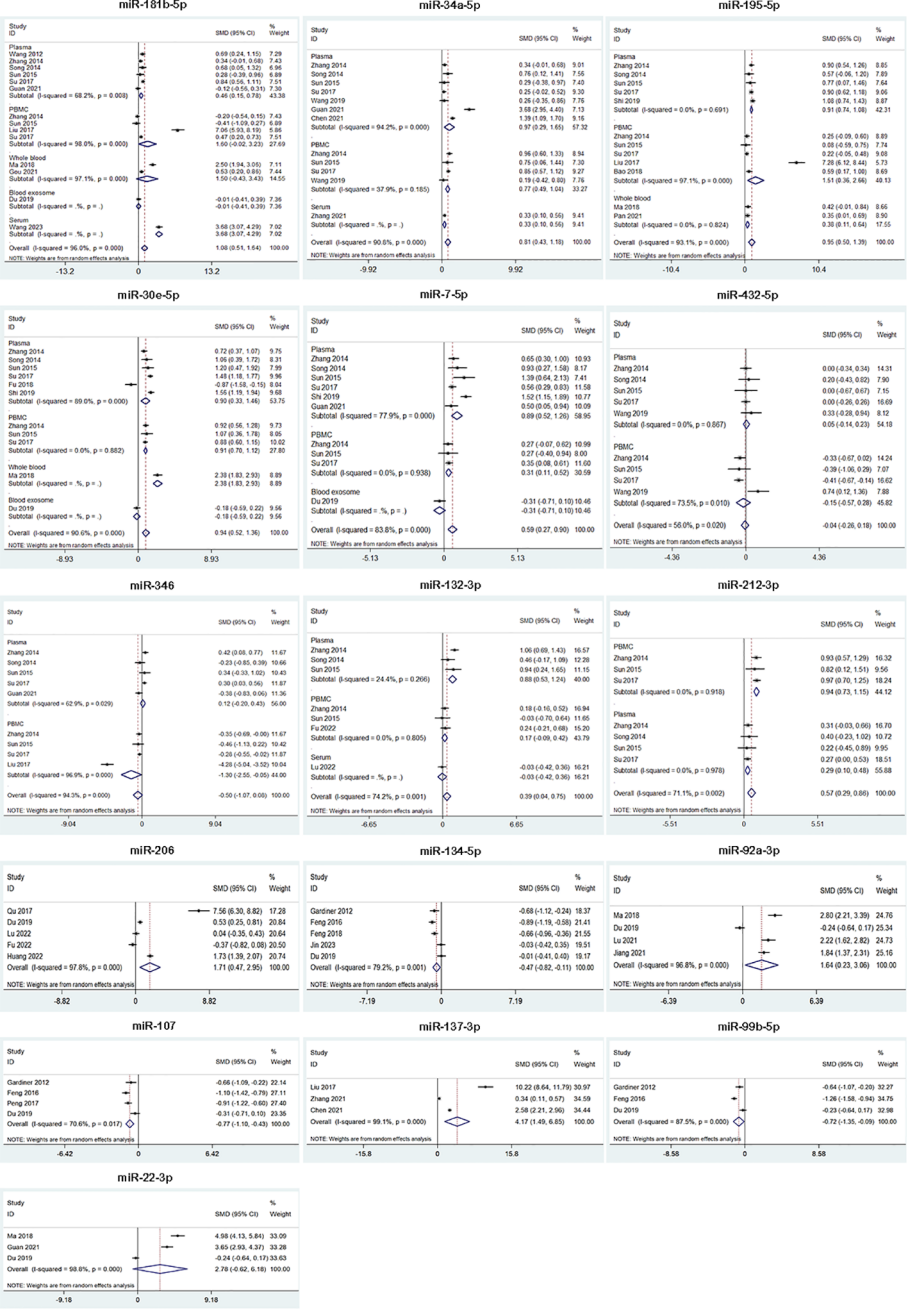
Forest plot of the meta-analysis of peripheral blood microRNAs of SZ patients versus controls.

In our meta-analysis, we analyzed the expression of 16 miRNAs from 29 studies for SZ. The results showed that SZ patients had higher miRNA levels than control group in miR-181b-5p, miR-34a-5p, miR-195-5p, miR-30e-5p, miR-7-5p, miR-132-3p, miR-212-3p, miR-206, miR-92a-3p and miR-137-3p, while lower miRNA levels than control group in miR-134-5p, miR-107, and miR-99b-5p. Besides, miR-432-5p, miR-346 and miR-22-3p were not dysregulated (**Table 2**, **Figure 2**). 9 of 16 miRNAs were included in subgroup analysis stratified by specimen type in SZ patients. The results revealed that miR-34a-5p, miR-30e-5p, miR-7-5p and miR-212-3p were both upregulated in plasma and PBMC. miR-195-5p was upregulated in plasma, PBMC and whole blood. miR-181b-5p was upregulated in plasma, but not in PBMC and whole blood. miR-132-3p was upregulated in plasma, but not in PBMC. miR-346 was downregulated in PBMC, but not in plasma. miR-432-5p was not dysregulated both in plasma and PBMC (**Figure 2**).

**Table 2:** Meta-analysis results of differentially expressed miRNAs in PDs reported in three or more studies.

Disease	miRNA	No. of study	Test of association			Test of heterogeneity			Direction	Publication bias	
			SMD(95*CI*)	*Z*	*P*	I^2^(%)	*P*	Model		Begg’s test *P*-Value	Egger’s test *P*-Value
SZ	miR-181b-5p	14	1.08(0.51,1.64)	3.73	1.93E-04	96.0	5.34E-60	R	up	0.324	0.066
	miR-34a-5p	12	0.81(0.43,1.18)	4.24	2.19E-05	90.8	2.81E-20	R	up	0.115	0.349
	miR-195-5p	12	0.95(0.50,1.39)	4.17	3.11E-05	93.1	2.26E-28	R	up	0.244	0.088
	miR-30e-5p	11	0.94(0.52,1.36)	4.38	1.21E-05	90.6	3.20E-18	R	up	0.876	0.736
	miR-7-5p	10	0.59(0.27,0.90)	4.65	2.42E-04	83.8	1.01E-08	R	up	0.371	0.577
	miR-432-5p	9	-0.04(-0.26,0.18)	0.37	7.12E-01	56.0	2.00E-02	R	/	0.754	0.167
	miR-346	9	-0.50(-1.07,0.08)	1.70	8.89E-02	94.3	1.80E-26	R	/	0.602	0.163
	miR-132-3p	7	0.39(0.04,0.75)	2.20	2.81E-02	74.2	7.26E-04	R	up	0.548	0.950
	miR-212-3p	7	0.57(0.29,0.86)	3.93	8.50E-05	71.1	2.01E-03	R	up	0.764	0.865
	miR-206	5	1.71(0.47,2.95)	2.70	6.95E-03	97.8	3.54E-38	R	up	1.000	0.286
	miR-134-5p	5	-0.47(-0.82,-0.11)	3.24	9.70E-03	79.2	7.17E-04	R	down	0.462	0.264
	miR-92a-3p	4	1.64(0.23,3.06)	2.27	2.30E-02	96.8	2.34E-20	R	up	0.308	0.097
	miR-107	4	-0.77(-1.10,-0.43)	4.53	5.97E-06	70.6	1.70E-02	R	down	0.734	0.201
	miR-137-3p	3	4.17(1.49,6.85)	3.05	2.29E-03	99.1	3.47E-51	R	up	0.296	0.216
	miR-99b-5p	3	-0.72(-1.35,-0.09)	2.25	2.46E-02	87.5	3.41E-04	R	down	1.000	0.363
	miR-22-3p	3	2.78(-0.62,6.18)	1.60	1.09E-01	98.8	3.36E-37	R	/	0.296	0.021
MDD	miR-124-3p	9	2.02(1.02,3.03)	3.96	7.62E-05	97.5	2.55E-65	R	up	0.348	0.174
	miR-16-5p	7	-0.79(-1.84,0.25)	1.49	1.37E-01	96.7	2.47E-36	R	/	0.368	0.776
	miR-132-3p	7	1.40(0.75,2.05)	4.23	2.29E-05	92.0	4.54E-14	R	up	0.133	0.335
	miR-155-5p	7	-0.64(-2.40,1.12)	0.71	4.76E-01	98.0	8.68E-64	R	/	0.548	0.846
	miR-139-5p	5	2.76(0.90,4.63)	2.91	3.65E-03	95.2	4.60E-17	R	up	0.806	0.585
	miR-451a	5	-0.94(-2.85,0.98)	0.96	3.37E-01	98.0	2.50E-41	R	/	0.806	0.932
	miR-146a-5p	5	-1.01(-2.22,0.21)	1.62	1.05E-01	97.1	9.05E-29	R	/	0.462	0.214
	miR-182-5p	4	3.72(1.64,5.79)	3.51	4.53E-04	97.4	8.85E-25	R	up	0.308	0.355
	miR-221-3p	4	2.72(1.72,3.73)	5.32	1.03E-07	93.2	1.35E-09	R	up	0.734	0.445
	miR-34a-5p	4	3.78(0.77,6.78)	2.46	1.38E-02	97.8	4.32E-29	R	up	0.734	0.633
	miR-145-5p	4	-0.15(-0.74,0.43)	0.51	6.07E-01	82.6	6.30E-04	R	/	0.734	0.500
	miR-144-5p	4	-1.65(-2.69,-0.60)	3.08	2.04E-03	93.5	6.18E-10	R	down	0.734	0.408
	miR-135a-5p	3	-10.65(-13.39,-7.90)	7.60	2.95E-14	87.1	4.29E-04	R	down	0.296	0.272
	miR-134-5p	3	-1.50(-3.43,0.44)	1.52	1.29E-01	98.0	6.65E-23	R	/	0.296	0.107
	miR-195-5p	3	-3.00(-6.90,0.90)	1.51	1.31E-01	98.9	2.11E-40	R	/	1.000	0.704
	miR-223-3p	3	0.25(-0.43,0.94)	0.72	4.72E-01	82.2	3.68E-03	R	/	1.000	0.459
	miR-93-5p	3	0.47(0.17,0.77)	3.11	1.88E-03	0.0	4.08E-01	F	up	0.296	0.154
	miR-21-5p	3	-0.18(-0.44,0.09)	1.30	1.93E-01	23.4	2.71E-01	F	/	1.000	0.592
	miR-106a-5p	3	0.17(-0.21,0.55)	0.88	3.81E-01	48.8	1.42E-01	F	/	1.000	0.525
	miR-126-3p	3	0.14(-0.19,0.47)	0.82	4.12E-01	0.0	6.92E-01	F	/	0.296	0.474
	let-7e-5p	3	0.00(-0.55,0.56)	0.01	9.91E-01	73.2	2.39E-02	R	/	1.000	0.648
	let-7b-5p	3	-0.21(-0.72,0.30)	0.80	4.24E-01	61.8	7.32E-02	R	/	1.000	0.813
	miR-17-5p	3	0.15(-0.39,0.70)	0.55	5.82E-01	72.4	2.66E-02	R	/	1.000	0.305
	miR-9-5p	3	1.18(-0.30,2.65)	1.57	1.17E-01	86.4	6.27E-04	R	/	1.000	0.637
	miR-26b-5p	3	-2.35(-7.36,2.65)	0.92	3.57E-01	99.3	1.24E-63	R	/	0.296	0.245
BD	miR-134-5p	4	-2.68(-5.63,0.28)	1.78	7.59E-02	99.3	4.45E-96	R	/	0.089	0.092
	miR-107	3	0.06(-0.24,0.35)	0.37	7.08E-01	60.3	8.04E-02	R	/	1.000	0.326

SZ, schizophrenia; MDD, major depression disorder; BD, bipolar disorder; SMD: standard mean difference; CI: confidence interval; R, random-effects model; F, fixed-effects model.

**Figure 3:**
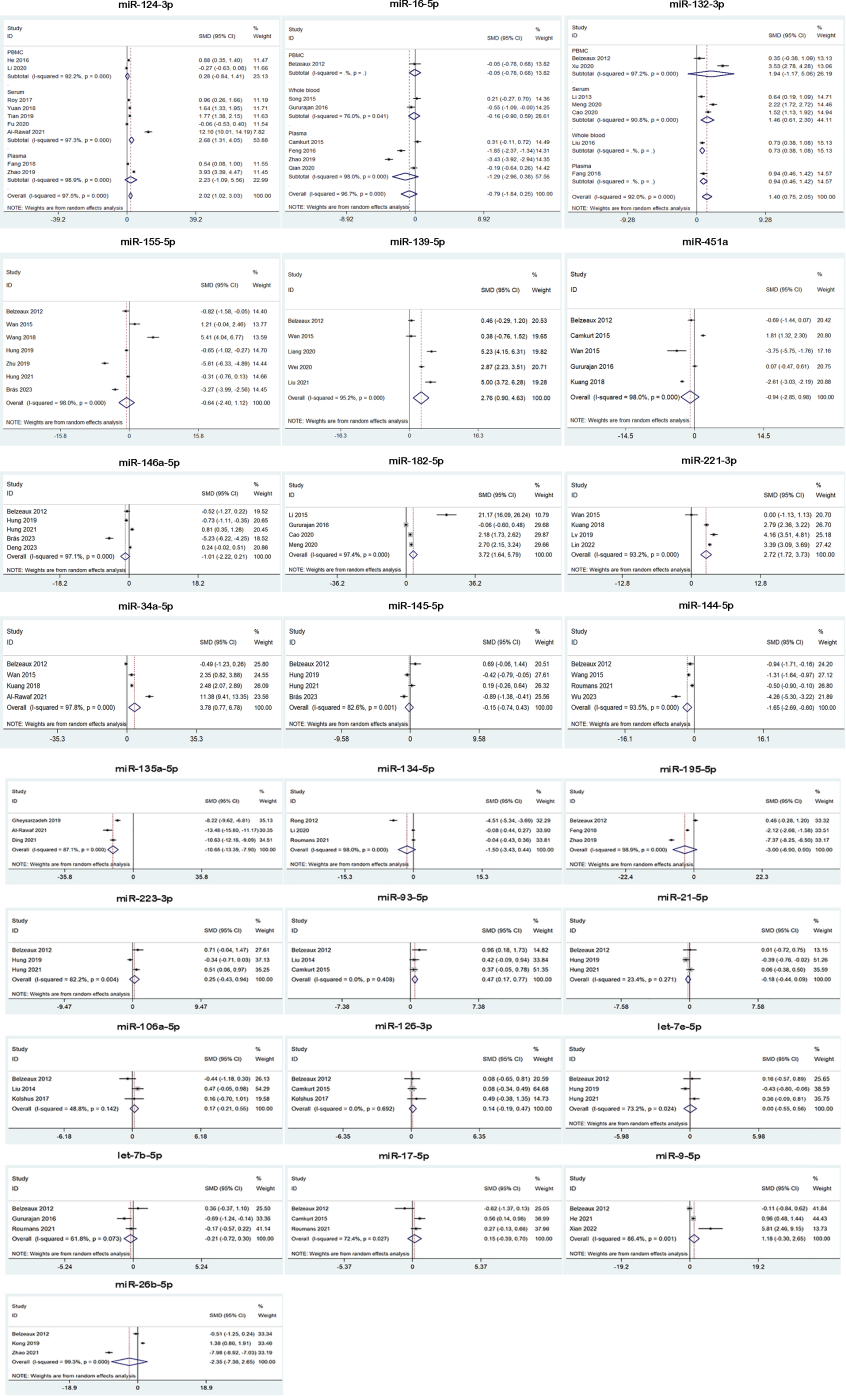
Forest plot of the meta-analysis of peripheral blood microRNAs of MDD patients versus control.

We analyzed the expression of 25 miRNAs from 45 studies for MDD. The results suggested that MDD patients had higher miRNA levels than control group in miR-124-3p, miR-132-3p, miR-139-5p, miR-182-5p, miR-221-3p, miR-34a-5p and miR-93-5p, while lower miRNA levels than control group in miR-144-5p and miR-135a-5p. Besides, miR-16-5p, miR-155-5p, miR-451a, miR-146a-5p, miR-145-5p, miR-134-5p, miR-195-5p, miR-223-3p, miR-21-5p, miR-106a-5p, miR-126-3p, let-7e-5p, let-7b-5p, miR-17-5p, miR-9-5p and miR-26b-5p were not dysregulated (**Table 2**, **Figure 3**). 3 of 25 miRNAs were included in subgroup analysis stratified by specimen type in MDD patients. The results showed that miR-124-3p was upregulated in serum, but not in plasma and PBMC. miR-16-5p was not dysregulated in plasma, whole blood and PBMC. miR-132-3p was upregulated in serum, plasma and whole blood, but not in PBMC. (**Figure 3**).

**Figure 4:**
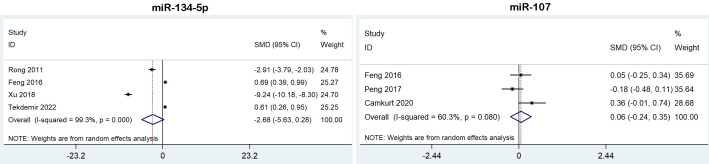
Forest plot of the meta-analysis of peripheral blood microRNAs of BD patients versus controls.

We analyzed the expression of miR-134-5p and miR-107 from 6 studies for BD, but neither of them were dysregulated in BD patients (**Table 2**, **Figure 4**).

### 3.3 Differentially expressed miRNAs in both SZ and MDD patients

Our results found that miR-132-3p and miR-34a-5p were upregulated in both SZ and MDD patients, suggesting that they may likely share some common molecular mechanisms.

### 3.4 Bioinformatics analysis

**Figure 5:**
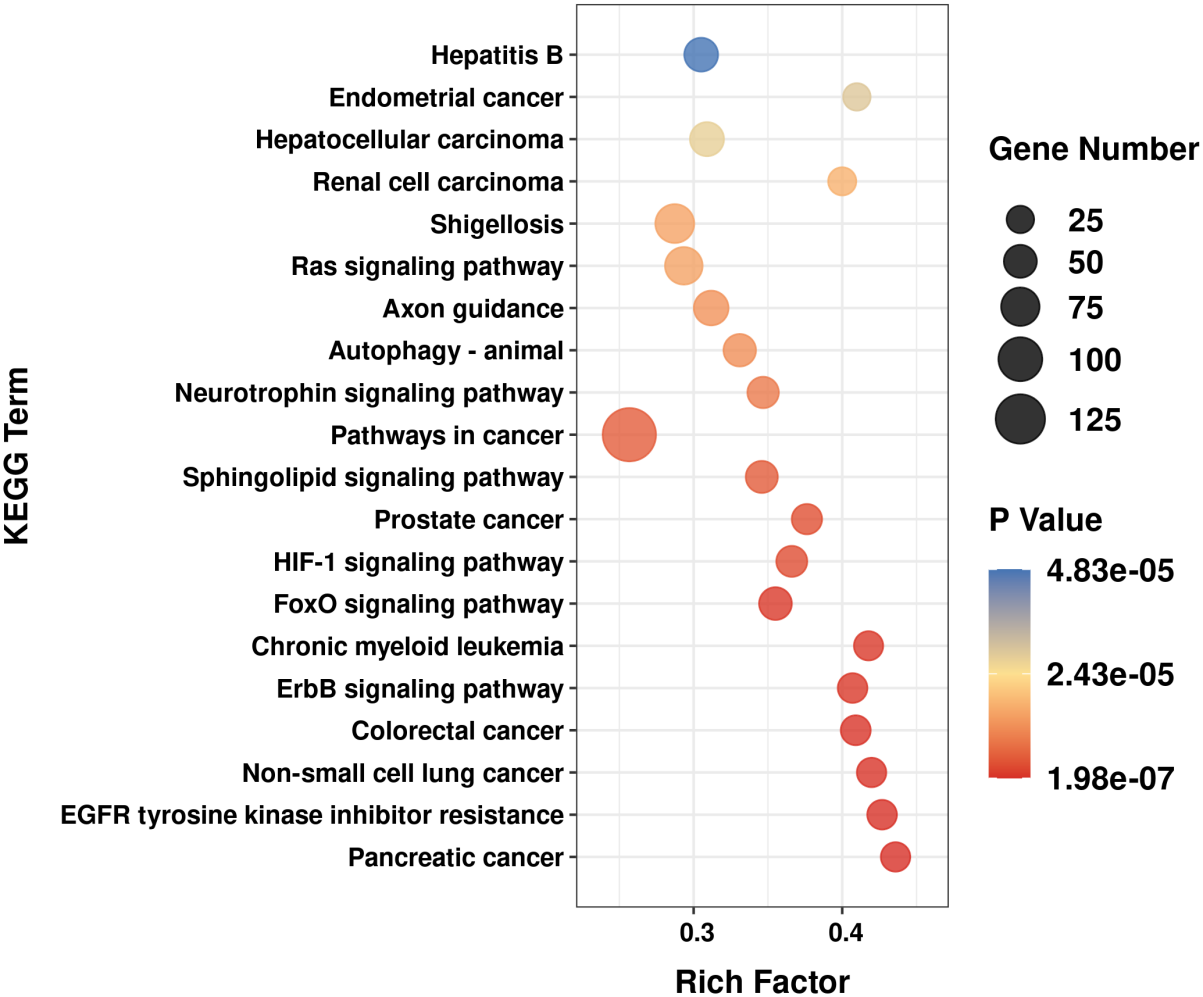
Top 20 significant enriched kyoto encyclopedia of genes and genomes (KEGG) signal pathway for the predicted target genes of miR-132-3p and miR-34a-5p.

To get insight into the possible roles of the miR-132-3p and miR-34a-5p, we performed target gene prediction and KEGG pathway analysis. A total number of 4138 target genes from miR-132-3p and miR-34a-5p were identified by using TargetScan and miRanda. **Figure 5** showed the top 20 significant enriched terms identified for KEGG pathway analysis, including axon guidance, neurotrophin signaling pathway, ErbB signaling pathway, FoxO signaling pathway, etc.

### 3.5 Publication bias and sensitivity analysis

Begg’s test and Egger’s test results indicated that there were no publication bias in this meta-analysis except miR-22-3p in SZ (**Table 2**). Sensitivity analysis showed that none of individual study could obviously influenced the pooled ORs except miR-22-3p in SZ, miR-223-3p and miR-17-5p in MDD (**Supplementary Figures 1**-**3**). For miR-22-3p, miR-223-3p and miR-17-5p, when Du et al.’s study, Huang et al.’s study, Belzeaux et al.’s were removed, respectively, the levels of these 3 miRNAs were all upregulated, but only 2 studies remained for each miRNA, so we wouldn’t further discuss them.

## 4 Discussion

A major goal of psychiatric research is to identify biomarkers for early and reliable diagnosis of PDs and guide their effective clinical treatment. In recent years, miRNAs, a key regulator of neurogenesis, neuronal differentiation, and synaptic plasticity, have received widespread attention as potential biomarkers of PDs (94, 95). However, as the literature reviews on miRNAs in PDs were merely narrative, or only meta-analysis for single disease (such as SZ or MDD), and no relatively comprehensive data was available. In this study we conducted a comprehensive and systematic meta-analysis for the first time to simultaneously identify dysregulated miRNAs expression profiles in SZ, MDD and BD.

In our meta-analysis, we focused on differentially expressed miRNAs derived from peripheral blood, excluding studies from brain tissue, as the method for extracting miRNAs from brain tissue has limited sample sources and are difficult to apply in clinical practice. Studies have found that brain disease-specific miRNAs can also be detected in peripheral blood, where their levels were highly correlated with those in the brain (96, 97). Interestingly, in SZ patients, miR-181b-5p and miR-132-3p were significantly increased in the pooled results, but only in certain blood elements in subgroup analysis based on specimen type. However, miR-346 was significantly decreased in PBMC, but not in plasma or the pooled results. In MDD patients, miR-124-3p was significantly increased in serum, but not in plasma and PBMC. These results indicated that the expression patterns of miRNAs could be affected by different specimen types. Previous evidence showed that the miRNA expression profiles in different blood elements may vary due to element-specific miRNAs released by specific tissues (98), unique miRNA features from unique lineage (99), different biological specimen processing conditions, and variation in reference miRNA levels (100).

With respect to SZ, Liu et al. (23) revealed that miR-181b-5p, miR-21-5p, miR-195-5p, miR-137, miR-346 and miR-34a-5p in PBMCs had high diagnostic sensitivity and specificity in SZ based on their meta-analysis of six diagnostic studies. Han et al. (101) found 27 significant differentially expressed miRNAs in SZ, of which 5 were downregulated, whereas 22 were upregulated. In our meta-analysis, we found the levels of miR-181b-5p, miR-34a-5p, miR-195-5p, miR-30e-5p, miR-7-5p, miR-132-3p, miR-212-3p, miR-206, miR-92a-3p and miR-137-3p were increased in SZ patients, while the levels of miR-134-5p, miR-107 and miR-99b-5p were decreased. Our research findings were not entirely consistent with the two previous meta-analysis, such as miR-195-5p was increased in SZ patients in our study but not in Han et al.’s study. The reasons for the inconsistent conclusion may be due to differences in inclusion and exclusion criteria, outcome measures, and the number of studies included. Of the thirteen differentially miRNAs in our study, miR-181b-5p was the most commonly reported one. Increased levels of miR-181b-5p have been detected in plasma (17, 19, 22), serum (43), as well as in brain of SZ patients (102). Of note, miR-181b-5p targeted a-amino-3-hydroxyl-5-methyl-4-isoxazolepropionate acid (AMPA) glutamate ionotropic receptor type subunit 2 (GRIA2) and the calcium sensor protein gene visinin like 1 (VSNL1) in SZ patients (102); both of these targets were themselves suspected to have a role in the pathology of SZ (103, 104). Guo et al. (105) constructed a miRNA-transcription factors regulatory network for SZ and found that miR-195-5p was one of the core regulators in this regulatory network. Many of the predicted target genes of miR-195-5p, such as regulator of G-protein signaling 4 (RGS4), N-methyl-D-aspartate (NMDA) glutamate ionotropic receptor type subunit 3A (GRIN3A), and reelin (RELN), have been reported to correlate with SZ (106, 107). Brain Derived Neurotrophic Factor (BDNF) was involved in neuronal plasticity, and multiple studies supported its close association with SZ (108). Mellios et al. showed that miR-195-5p regulated BDNF, thereby affecting the expression of downstream gamma-aminobutyric acid (GABA)ergic transcripts, such as parvalbumin (PV), somatostatin (SST), and neuropeptide Y (NPY) in SZ (109, 110). Xu et al. (111) indicated that a potentially functional variant that affected pre-miR-30-5p played a role in SZ susceptibility. Overexpression of miR-30e-5p in the rat brain could lead to cognitive impairment, resulting in anxiety, depression, and SZ like symptoms (112). Abnormal expression of miR-7-5p could inhibit the protein kinase AKT1 gene, which has been confirmed to be a susceptibility gene for SZ (113). In addition, Zhang et al. (114) found that miR-7-5p was overexpressed in plasma of SZ and the overexpression of miR-7-5p significantly inhibited the expression levels of SH3 and multiple ankyrin repeat domains protein 3 (SHANK3), which in turn may play an essential role in the pathological process of SZ. It was found that miR-212-3p was co-transcribed with miR-132-3p, the miR-132-3p/miR-212-3p family influenced genes associated with circadian clock entrainment (115), which was consistent with the defective circadian synchronization observed in SZ. A recent study suggested that miR-206 may contribute to SZ risk through allele-dependent regulation of the genome-wide significant gene NT5C2 (116). Du et al. (32) showed significantly increased miR-206 levels and decreased BDNF levels in SZ, and antipsychotics restored the dysregulations of miR-206 and BDNF in SZ, suggesting that upregulation of miR-206 may contribute to the dysfunction of BDNF in SZ. miR-92a-3p was related to synaptic transmission (117). Studies have confirmed that miR-137-3p was closely related to the development and maturation of the nervous system, and can regulate multiple neural development signaling pathways and target gene expression through cascade effects (118). Wright et al. (119) identified the possible regulatory signaling pathways involved in SZ by miR-137-3p through functional enrichment analysis, including axonal guidance, Ephrin receptor signaling, long-term regulation, Sertoli cell junction, and protein kinase A signaling. Kwon et al. (120) confirmed that susceptibility genes of SZ, such as transcription factor 4 gene (TCF4), calcium voltage-gated channel subunit alpha1 C gene (CACNA1C), CUB, and Sushi multiple domains 1 gene (CSMD1), WW domain binding protein 1 like gene (C10orf26) were target genes for miR-137-3p. miR-134-5p was a brain-specific miRNA that presented in the synaptic dendrite chamber of hippocampal neurons, which repressed dendritic spine size by inhibiting the translation of Lim kinase 1 (Limk1) mRNA, thereby affecting the strength of excitatory synapses (121). More recently, it has been shown that the expression of silent information regulator 1 (SIRT1), which modulates synaptic plasticity and memory formation, is regulated by cAMP-response element-binding protein (CREB), which itself is translationally repressed by miR-134-5p (121, 122). Beveridge et al. (107) suggested that miR-107 were highly enriched in pathways involved in neural connectivity and synaptic plasticity, such as axon guidance, long-term potentiation. Scarr et al. (123) demonstrated that miR-107 could regulate the expression of cortical muscarinic M1 receptors (CHRM1), which was involved in the pathophysiology of SZ (124). Kaurani et al. (125) reported that miR-99b-5p regulated Z-DNA binding protein 1 (Zbp1) to control inflammatory response in microglia, which may contributed to the pathogenesis of SZ.

Regarding MDD, Li et al. (126) showed that 17 miRNAs had high sensitivity and specificity in diagnosing MDD based on 7 studies. We found the levels of miR-124-3p, miR-132-3p, miR-139-5p, miR-182-5p, miR-221-3p, miR-34a-5p and miR-93-5p were increased, while the level of miR-144-5p and miR-135a-5p were decreased. Our research findings were not entirely consistent with Li et al.’s meta-analysis, such as miR-16-5p was not dysregulated in MDD patients in our study but was upregulated in their study. The most possible reason for the inconsistent conclusion may be their meta-analysis based on diagnostic studies and all mentioned miRNAs only reported in single study, but our present study included miRNAs from at least 3 independent studies. Moreover, we conducted subgroup analysis stratified by specimen type. Of the seven differentially expressed miRNAs in our study, miR-124-3p was the most frequently reported one. Increased levels of miR-124-3p have been consistently detected in serum (58, 79) and plasma (60, 69). miR-124-3p was a rich brain-specific miRNA that inhibited serotonin induced synaptic facilitation by regulating CREB, thereby negatively regulating synaptic plasticity (127). Studies indicated that miR-124-3p could inhibit the expression of BDNF in the hippocampus of depression model rats (128). BDNF was a validated miR-124-3p target (47) and low expression levels of BDNF played a predominant role in the pathophysiology of MDD (129). miR-139-5p might act as a negative regulator for neural stem cell proliferation and neuronal differentiation, and modulated cortical neuronal migration by targeting lissencephaly-1 (Lis1) (77, 130). Wei et al. (77) found stress-induced elevation of miR-139-5p caused impairment of hippocampal neurogenesis and depressive-like behaviors in adult mice. miR-182-5p has been proven to be an important regulatory factor in the nervous system, involved in various biological processes such as neuronal survival (131), axonogenesis (132), and protein signal transduction (133). Studies have provided evidence for miR-182-5p as a modulator of the endogenous circadian clock (134). Disruption of circadian rhythms has long been implicated in the pathophysiology of MDD (135). Li et al. (47) found the serum levels of miR-182-5p were increased and BDNF levels were reduced in MDD patients, which supported that miR-182-5p could negatively regulate BDNF expression and might be related to the development of MDD. Although miR-221-3p was commonly considered as a tumor regulator, in recent years, some researchers have been repeatedly reported abnormally high levels of miR-221-3p in the cerebrospinal fluid (CSF) and serum of MDD patients (49, 136), suggesting that miR-221-3p may also be involved in the pathogenesis of MDD. Studies revealed that miR-221-3p was closely related to neuronal development and axon growth (137, 138). In addition, Lian et al. (139) demonstrated that miR-221-3p could promote the development of MDD by modulating Wnt2/CREB/BDNF axis. Wu et al. (89) demonstrated that miR-144-5p influenced synaptic plasticity by targeting phosphatase and tensin homolog (PTEN), and miR-144-5p exerted anti-inflammatory effects in patients with MDD. miR-135a-5p regulated axon growth/regeneration and mediated long-term depression (140, 141). Ding et al. (78) demonstrated that miR−135a-5p regulated apoptosis and inflammatory response in mouse hippocampal neurons by regulating the expression of Toll like receptor 4 (TLR 4), thereby alleviating the depressive behavior of mice and playing a protective role in depression. Valiuliene et al. (142) revealed that miR-93-5p may regulate the expression of the pro-inflammatory cytokine IL-18, involving in the pathophysiology of MDD.

Our results found that miR-132-3p and miR-34a-5p were increased in both SZ and MDD patients, suggesting that they may likely share some common molecular mechanisms. miR-132-3p was a miRNA enriched in the brain and participated in axonal growth, proliferation and synaptic plasticity (115). Neuronal plasticity and its related pathways have shown to be disturbed in SZ and MDD (143, 144). miR-132-3p targeted important genes that regulate neuronal plasticity, including BDNF, methyl-CpG-binding protein 2 (MeCP2), GTPase activating protein (p250GAP) (145–147). Su et al. (148) demonstrated that miR−132-3p was significantly increased in the peripheral blood of MDD patients, while BDNF and MeCP2 were decreased, and the level of miR-132-3p was negatively correlated with the protein expression levels of MeCP2 and BDNF. Low BDNF level was also detected in CSF and plasma of SZ patients (149). Besides, MeCP2 has been repeatedly reported as a risk gene for SZ (150, 151). p250GAP was a brain-enriched NDMA receptor-interacting RhoGAP. Studies have shown that the p250GAP gene was associated with risk for SZ and MDD (152, 153). miR-34a-5p suppressed SIRT1, leading to increased acetylated p53, a regulator of the cell cycle progression and cellular senescence (154). It has also been shown that miR-34a-5p was a transcriptional target of p53, thus establishing a positive feedback loop between miR-34a-5p, p53, and SIRT1 (154, 155). Oxidative stress induced the upregulation of p53 activity, consequently increasing the expression levels of miR-34a-5p (155, 156). Both SZ and MDD were associated with high oxidative stress levels (157, 158), which could elucidate the upregulated miR-34a-5p found in these patients. In addition, SZ and MDD also were genetically associated with the SIRT1 gene (159–161). Xu et al. (162) indicated that miR-34a-5p targeted the NMDA receptors (including Grin1, Grin2a, and Grin2b), providing evidence of a post-transcriptional mechanism of SZ and MDD associated glutamatergic and synaptic dysfunction (163–165). Moreover, KEGG pathway analysis in the present study indicated that the identified signaling pathways enriched by the predicted target genes of miR-132-3p and miR-34a-5p, such as axon guidance, neurotrophin signaling pathway, ErbB signaling pathway, FoxO signaling pathway, were closely related to the pathologic mechanisms of SZ and MDD (166–171). Interestingly, enriched KEGG pathways also contained cancer pathways, which may be involved in shared pathogenesis of SZ and MDD. For example, PI3K/Akt pathway, which modulated by miR-132-3p, was a prototypic cancer pathway (172). Many genes on PI3K/Akt pathway were considered to be potentially susceptible genes for the development of SZ (173). The levels of Akt1 were decreased in the brain, as well as in the peripheral lymphocytes of individuals with SZ (174). P13K/Akt signaling cascade also was strongly linked with the neurobiology of MDD (175). Reduced Akt1 activity was found in the brain of MDD patients (176). Evidence showed that p53, which could regulate the transcription of miR-34a-5p, was one of the most important tumor suppressor genes (177). Catts et al. (178) proposed that p53 might be a candidate susceptibility gene for SZ by regulating apoptosis. Mahmood et al. (179) suggested the protective effect of minor allele 72C of p53 gene towards MDD.

The following limitations of the study should be considered. Firstly, between-study heterogeneity remained substantial although we performed subgroup analyses to explore their sources. The possible causes of heterogeneity may be related to the duration, severity, and treatment of patient’s disease. Due to the limited information provided by the included studies, further analysis was not possible. Secondly, the majority of the population included in the study came from China, which may limit the broad applicability of the findings. Thirdly, potential publication bias may affect the present results due to the relative small number of studies included for some miRNAs. Finally, most of miRNAs included in the present meta-analysis were detected by qPCR, which may also result in bias.

## 5 Conclusion

In summary, our study identified 13 differentially expressed miRNAs in SZ, 9 differentially expressed miRNAs in MDD, among which miR-132-3p and miR-34a-5p were upregulated in both SZ and MDD by systematically analyzing qualified studies. These miRNAs may be used as potential biomarkers for the diagnosis of SZ and MDD in the future. Further validation in large patient cohorts is required to confirm the findings.

## Data availability statement

The original contributions presented in the study are included in the article/**Supplementary Material**, further inquiries can be directed to the corresponding author.

## Author contributions

XL: Conceptualization, Data curation, Formal Analysis, Validation, Writing – original draft. LD: Data curation, Formal Analysis, Investigation, Methodology, Writing – original draft. ZJ: Investigation, Resources, Validation, Writing – original draft. MS: Software, Validation, Visualization, Writing – review & editing. PY: Conceptualization, Funding acquisition, Project administration, Supervision, Writing – original draft, Writing – review & editing.
