# Supplementary material for: Identifying the differentially expressed peripheral blood microRNAs in psychiatric disorders: a systematic review and meta-analysis
Source: Front Psychiatry. 2024 May 17;15:1390366. doi: 10.3389/fpsyt.2024.1390366 (PMC11140110; doi:10.3389/fpsyt.2024.1390366)
Supplement: Supplementary file 1 [file DataSheet_1.docx]

**Supplementary Materials**

| **Number** | **Author** | **DOI** |
| --- | --- | --- |
| 1 | Lai 2011 | 10.1371/journal.pone.0021635 |
| 2 | Shi 2012 | 10.1016/j.jpsychires.2011.09.010 |
| 3 | Gardiner 2012 | 10.1038/mp.2011.78 |
| 4 | Liu 2014 | 10.1016/j.jad.2013.12.035 |
| 5 | Issler 2014 | 10.1016/j.neuron.2014.05.042 |
| 6 | Fan 2014 | 10.1016/j.jpsychires.2014.08.007 |
| 7 | Wei 2015 | 10.1176/appi.ajp.2015.14030273 |
| 8 | Yu 2015 | 10.1016/j.pnpbp.2015.05.007 |
| 9 | Lai 2016 | 10.1038/tp.2015.213 |
| 10 | Maffioletti 2016 | 10.1016/j.jad.2016.04.021 |
| 11 | Kolshus 2017 | 10.1111/acps.12821 |
| 12 | Ma 2018 | 10.1016/j.psychres.2018.03.080 |
| 13 | Geaghan 2019 | 10.1016/j.jpsychires.2019.02.023 |
| 14 | Fries 2019 | 10.1016/j.jad.2019.05.020 |
| 15 | Zhao 2019 | 10.1038/s41398-019-0609-0 |
| 16 | Zhang 2020 | 10.1038/s41398-020-0773-2 |
| 17 | Chen 2020 | 10.3389/fpsyt.2020.00757 |
| 18 | Lee 2020 | 10.1038/s41598-020-58195-0 |
| 19 | You 2020 | 10.3892/mmr.2020.11513 |
| 20 | Tabano 2020 | 10.1007/s00406-019-01057-2 |
| 21 | Liu 2021 | 10.1016/j.neulet.2021.136184 |
| 22 | Gecys 2022 | 10.1155/2022/3004338 |
| 23 | Tabano 2022 | 10.1007/s00406-019-01057-2 |
| 24 | Jin 2023 | 10.1016/j.jpsychires.2023.01.035 |

**Table 1**  The studies for which didn't receive a response

| **Number** | **Disease** | **Author** | **DOI** |
| --- | --- | --- | --- |
| 1 | SZ | Peng 2014 | 10.16571/j.enki.1008-8199.2014.12.022 |
| 2 |  | Peng 2015 | CNKI:SUN:LCJS.0.2015-01-032 |
| 3 |  | Wei 2015 | 10.1176/appi.ajp.2015.14030273 |
| 4 |  | Xu 2016 | 10.1093/schbul/sbv170 |
| 5 |  | Zhao 2019 | 10.1038/s41398-019-0609-0 |
| 6 |  | Yu 2019 | 10.3760/cma.j.issn.1008-1372.2019.11.028 |
| 7 |  | Zhang 2019 | 10.16680/j.1671-3826.2019.09.29 |
| 8 |  | You 2020 | 10.3892/mmr.2020.11513 |
| 9 |  | Lei 2021 | 10.3969/j.issn.1009-6469.2021.06.035 |
| 10 |  | Davarinejad 2022 | 10.1007/s12031-021-01945-0 |
| 11 |  | Xu 2022 | 10.13602/j.cnki.jels.2022.12.12 |
| 12 |  | Ni 2023 | 10.3969/j.issn.1006-1959.2023.02.013 |
| 13 | MDD | Fan 2014 | 10.1016/j.jpsychires.2014.08.007 |
| 14 |  | Sun 2015 | 10.1016/j.jad.2016.01.017 |
| 15 |  | Maffioletti 2016 | 10.1016/j.jad.2016.04.021 |
| 16 |  | Wan 2018 | 10.3969/j.issn.1009-5519.2018.24.011 |
| 17 |  | Zhao 2019 | 10.1016/j.jad.2018.10.363 |
| 18 |  | Mendes-Silva 2019 | 10.1016/j.jpsychires.2019.01.019 |
| 19 |  | Yu 2019 | 10.3760/cma.j.issn.1006-7884.2019.02.006 |
| 20 |  | Li 2021 | 0.1186/s12974-020-02040-8 |
| 21 |  | Liu 2021 | 10.1016/j.neulet.2021.136184 |
| 22 |  | Li 2021 | 10.3969/j.issn.1672-187X.2021.01.005 |
| 23 |  | Meng 2021 | 10.3760/cma. j. cn311847-20210430-00138 |
| 24 |  | Ran 2022 | 10.17305/bjbms.2022.7110 |
| 25 |  | Ho 2023 | 10.1016/j.comppsych.2022.152363 |
| 26 |  | Wang 2023 | 10.1016/j.jad.2023.05.068 |
| 27 |  | Jiang 2023 | 10.3969/j.issn.1007-3205.2023.01.008 |
| 28 | BD | Wang 2011 | 10.3969/j. issn. 1002-0829. 2011.05.003 |
| 29 |  | Liao 2019 | CNKI:SUN:LCJS.0.2019-06-020 |
| 30 |  | Tabano 2020 | 10.1007/s00406-019-01057-2 |
| 31 |  | Lee 2020 | 10.1038/s41598-020-58195-0 |
| 32 |  | Ceylan 2020 | 10.1016/j.jad.2019.10.038 |
| 33 |  | Chen 2020 | 10.3389/fpsyt.2020.00757 |
| 34 |  | Tekin 2022 | 10.47626/1516-4446-2021-2260 |
| 35 |  | Bella 2023 | 10.3390/genes14091778 |

**Table 2** The studies excluded due to the “minimum three studies rule”

**
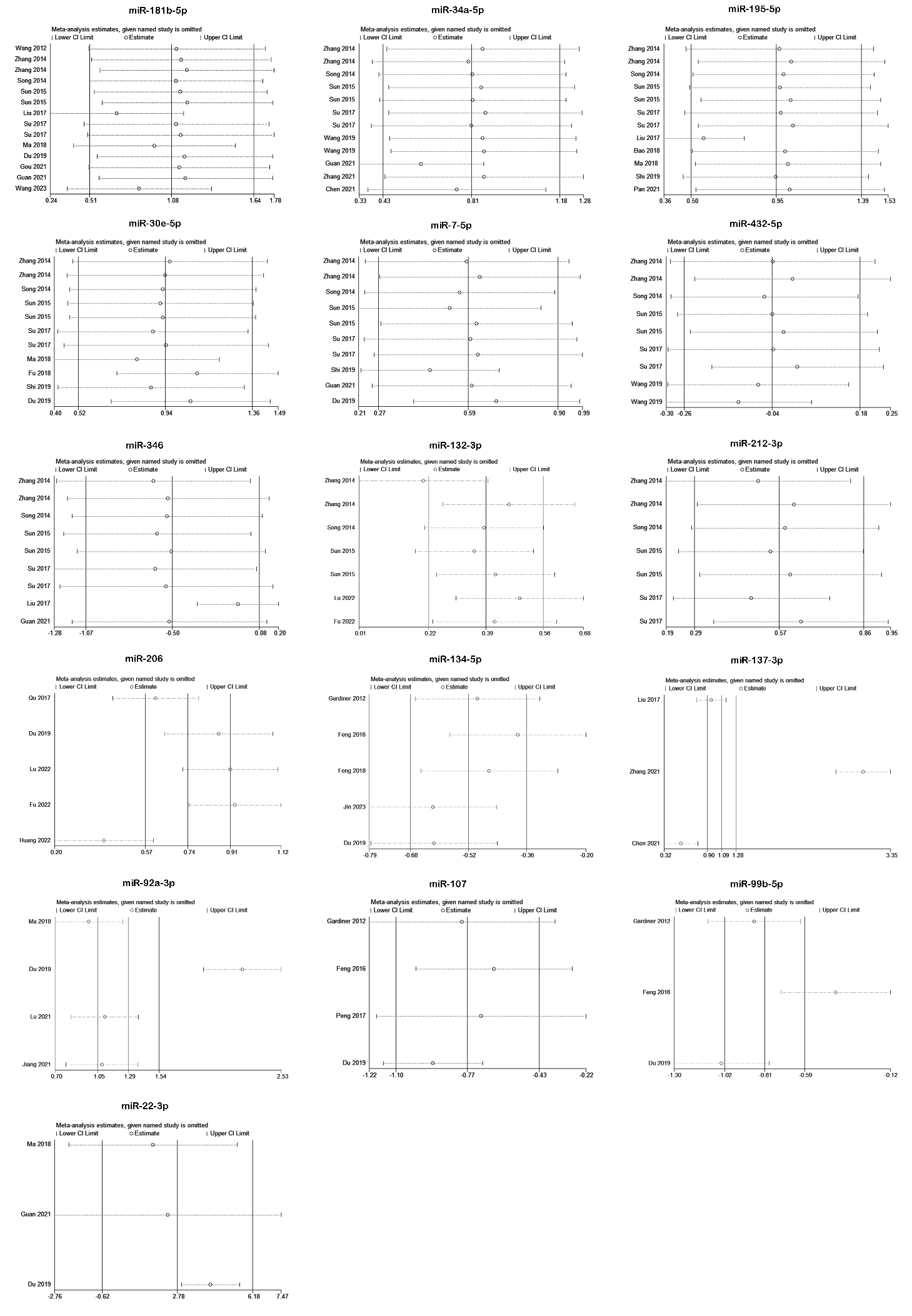
**

**Figure 1** Sensitivity analysis of the meta-analysis of peripheral blood microRNAs of SZ patients versus controls.


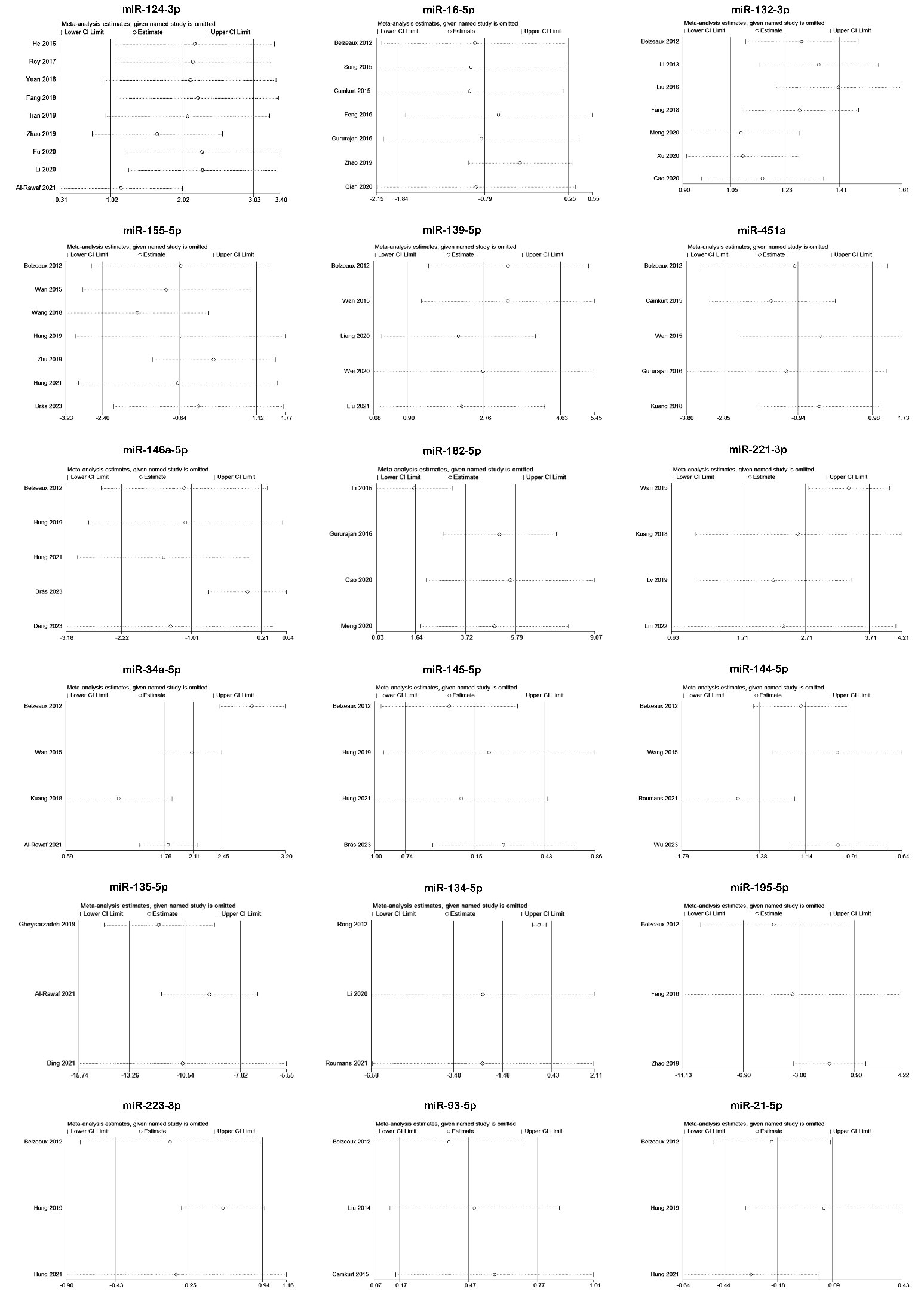


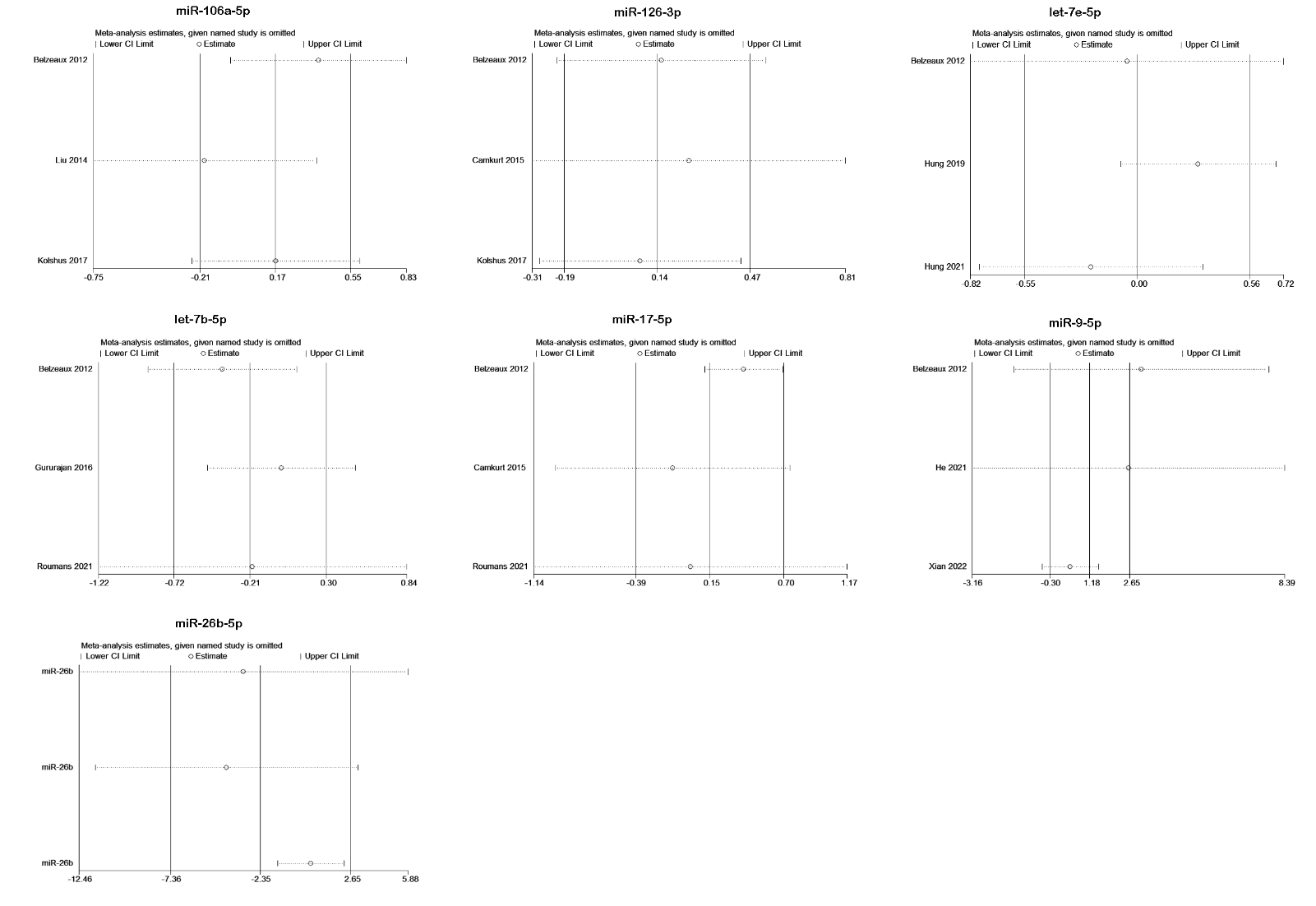


**Figure 2** Sensitivity analysis of the meta-analysis of peripheral blood microRNAs of MDD patients versus controls.


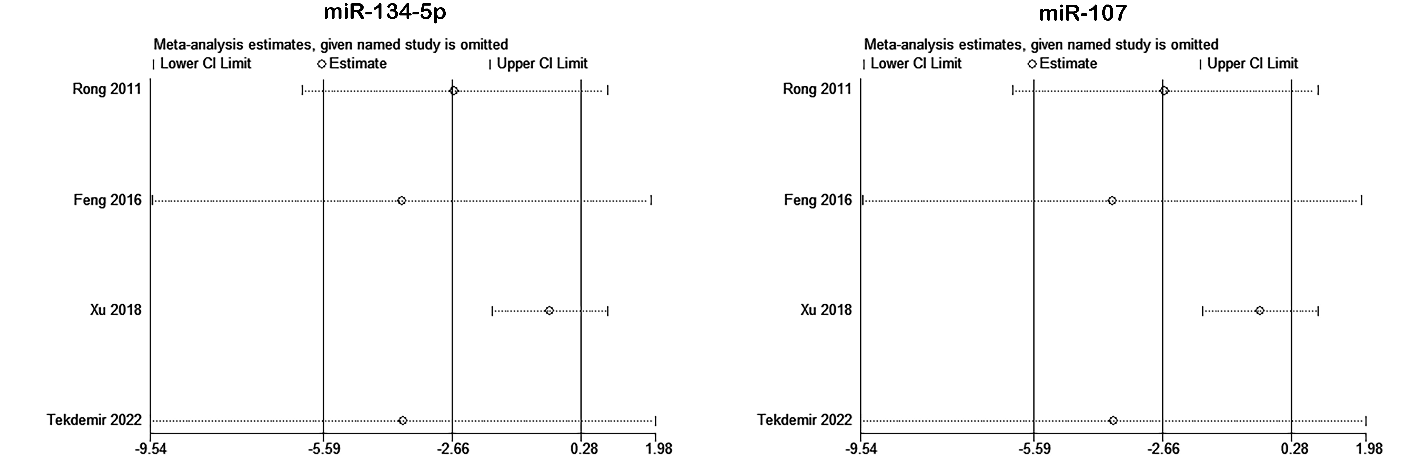


**Figure 3** Sensitivity analysis of the meta-analysis of peripheral blood microRNAs of BD patients versus controls.
